# Indigenous Arbuscular Mycorrhizal Fungal Assemblages Protect Grassland Host Plants from Pathogens

**DOI:** 10.1371/journal.pone.0027381

**Published:** 2011-11-16

**Authors:** Jeannine Wehner, Pedro M. Antunes, Jeff R. Powell, Tancredi Caruso, Matthias C. Rillig

**Affiliations:** 1 Dahlem Center of Plant Sciences, Plant Ecology, Institut für Biologie, Freie Universität Berlin, Berlin, Germany; 2 Department of Biology, Algoma University, Sault Ste. Marie, Ontario, Canada; 3 Hawkesbury Institute for the Environment, University of Western Sydney, Penrith, New South Wales, Australia; Nanjing Agricultural University, China

## Abstract

Plant roots can establish associations with neutral, beneficial and pathogenic groups of soil organisms. Although it has been recognized from the study of individual isolates that these associations are individually important for plant growth, little is known about interactions of whole assemblages of beneficial and pathogenic microorganisms associating with plants.

We investigated the influence of an interaction between local arbuscular mycorrhizal (AM) fungal and pathogenic/saprobic microbial assemblages on the growth of two different plant species from semi-arid grasslands in NE Germany (Mallnow near Berlin). In a greenhouse experiment each plant species was grown for six months in either sterile soil or in sterile soil with one of three different treatments: 1) an AM fungal spore fraction isolated from field soil from Mallnow; 2) a soil pathogen/saprobe fraction consisting of a microbial community prepared with field soil from Mallnow and; 3) the combined AM fungal and pathogen/saprobe fractions. While both plant species grew significantly larger in the presence of AM fungi, they responded negatively to the pathogen/saprobe treatment. For both plant species, we found evidence of pathogen protection effects provided by the AM fungal assemblages. These results indicate that interactions between assemblages of beneficial and pathogenic microorganisms can influence the growth of host plants, but that the magnitude of these effects is plant species-specific.

## Introduction

Under natural conditions plant roots interact with different soil organisms, which can be beneficial, neutral or pathogenic. The role of these interactions has been increasingly recognized [e.g.], [ 1]–[3]. Beneficial organisms like arbuscular mycorrhizal (AM) fungi may influence plant community structure, diversity [4], [5] and productivity [6], [7] in a natural ecosystem. Pathogenic and parasitic soil organisms, such as viruses, bacteria, insects or fungi, have been also observed to promote plant diversity and significantly determine plant community composition [8].

AM fungi are the most common symbiotic fungi, associated with many different plant species [9] and completely dependent on their host's carbon. In return the plant receives additional nutrients [10], improved water relations [11] and pathogen protection [e.g.], [ 12]–[15] from AM fungi. Several mechanisms whereby AM fungi could cause pathogen protection are known, including changes in root architecture, improved nutrient status, activation of plant defense mechanisms, or competition for infection sites [16]. However, most studies on AM fungal mediated pathogen protection were carried out with selected, usually single, isolates of both pathogenic and AM fungi. For instance, Newsham et al. [12] used one *Glomus* sp. as AM fungal treatment and *Fusarium oxysporum* as pathogen treatment. While the AM fungus provided protection against *Fusarium*, it did not increase the nutrient status of the host plant. In natural ecosystems a mycorrhizal plant species is associated not only with one AM fungus but with an entire AM fungal assemblage [17], and, as a consequence, community-level emergent properties in determining the outcomes of AM fungal-pathogen interactions are poorly understood [18]. For example, AM fungi may display functional complementarity in terms of protecting the host plant from a pathogen [18]: while one species might provide nutrients to their host, another one might increase the tolerance or resistance against pathogens, both leading to protection from the pathogen. Therefore, it might be important to consider AM fungal assemblages in the context of pathogen protection. However, not only AM diversity can influence pathogen protection, but also the assemblage of pathogens may need to be considered. It is known that host plants are often exposed to several pathogens simultaneously [19], but there are only relatively few studies with multiple pathogens approaches [e.g.], [ 20]–[22].

Here we investigated the interaction of local AM fungal and soil pathogen/saprobe assemblages for two different plant species. Building on work on single isolates, we hypothesized that a natural AM fungal assemblage would provide protection against soil pathogen/saprobe assemblages.

## Results

### Performance of the plant species

**Figure 1:**
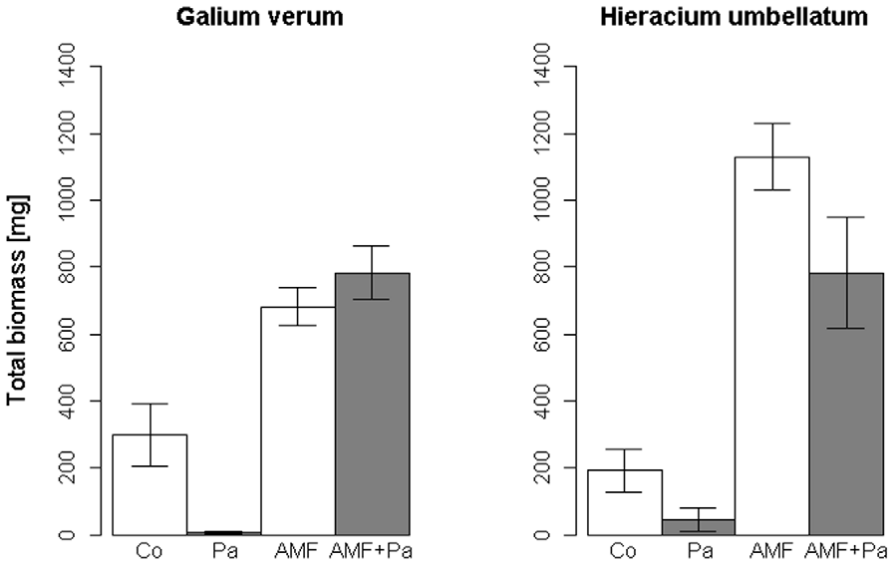
Effect of indigenous soil microbial treatment on the total biomass of *Galium verum* and *Hieracium umbellatum* in the four treatments (Co = Control, Pa = Pathogen community, AMF = AM fungal community, AMF + Pa). Error bars represent the standard error of the mean.

We generally found significant AM fungal and pathogen effects as well as a significant interaction in both *Hieracium umbellatum* and *Galium verum* (see MANOVAs; Tables 1 and 2). More specifically, individuals of both species inoculated with the AM fungal fraction had a significantly higher total biomass than those without AM fungi (Fig. 1). In contrast, inoculation with the pathogen/saprobe fraction significantly reduced total biomass. While *G. verum* responded negatively to pathogen inoculation only in the absence of AM fungi (Table 3), *H. umbellatum* showed this response independent of AM fungal inoculation (Table 4).

**Table 1:** Multivariate analysis of variance for *Hieracium umbellatum* for the response variables total biomass, fine root length, coarse root length and root diameter.

	Df	Pillai	Approx F	Num Df	Den Df	Pr(>F)
myco	1	0.93947	50.442	4	13	<0.0001***
patho	1	0.77373	11.113	4	13	0.0003**
myco∶patho	1	0.58083	4.503	4	13	0.015*

(* = p<0.05; ** p<0.001; *** = p<0.0001, myco = AM fungal treatment, patho = Pathogen treatment, myco∶patho = Interaction of AM fungal and pathogen treatment).

**Table 2:** Results from the Multivariate analysis of variance for *Galium verum* for the response variables total biomass, fine root length, coarse root length and root diameter.

	Df	Pillai	Approx F	Num Df	Den Df	Pr(>F)
myco	1	0.91414	34.602	4	13	<0.0001***
patho	1	0.73940	9.221	4	13	0.0009**
myco∶patho	1	0.82527	15.351	4	13	<0.0001***

(* = p<0.05; ** p<0.001; *** = p<0.0001, myco = AM fungal treatment, patho = Pathogen treatment, myco∶patho = Interaction of AM fungal and pathogen treatment).

**Table 3:** Results from analyses of variance on different response variables for *Galium verum*.

	Effect	Df	Sum Sq	F-value	p-value
Total biomass	myco	1	413946	49.4803	<0.0001***
	patho	1	3172	0.3792	0.5467
	myco∶patho	1	85017	10.1624	0.006*
	Residuals	16	130.4		
Fine root length(<2 mm)	myco	1	9433182	42.7560	<0.0001***
	patho	1	507064	2.2983	0.1490
	myco∶patho	1	5043393	22.8592	0.0002**
	Residuals	16	3530055		
Coarse root length(>2 mm)	myco	1	1296085	117.4398	<0.0001***
	patho	1	60160	5.4512	0.03291*
	myco∶patho	1	78746	7.1353	0.0167*
	Residuals	16	176579		
Root diameter	myco	1	0.8	0.1074	0.7474
	patho	1	192.2	25.7987	<0.0001***
	myco∶patho	1	352.8	47.3557	<0.0001***
	Residuals	16	119.2		

(* = p<0.05; ** p<0.001; *** = p<0.0001, myco = AM fungal treatment, patho = Pathogen treatment, myco∶patho = Interaction of AM fungal and pathogen treatment).

**Table 4:** Results from analyses of variance on different response variables for *Hieracium umbellatum*.

	Effect	Df	Sum Sq	F-value	p-value
Total biomass	myco	1	500.0	109.4391	<0.0001***
	patho	1	88.2	19.3051	0.0004**
	myco∶patho	1	3.2	0.7004	0.4150
	Residuals	16	73.1		
Fine root length(<2 mm)	myco	1	3162235	75.0230	<0.0001***
	patho	1	611228	14.5012	0.0015*
	myco∶patho	1	47981	1.1383	0.3018
	Residuals	16	674403		
Coarse root length(>2 mm)	myco	1	969.36	67.4333	<0.0001***
	patho	1	200.21	13.9275	0.0018*
	myco∶patho	1	46.48	3.2335	0.0910.
	Residuals	16	230.0		
Root diameter	myco	1	16.2	0.9818	0.3365
	patho	1	180.0	10.9091	0.0045*
	myco∶patho	1	204.8	12.4121	0.0028*
	Residuals	16	264.0		

(* = p<0.05; ** p<0.001; *** = p<0.0001, myco = AM fungal treatment, patho = Pathogen treatment, myco∶patho = Interaction of AM fungal and pathogen treatment).

### Root morphology

**Figure 2:**
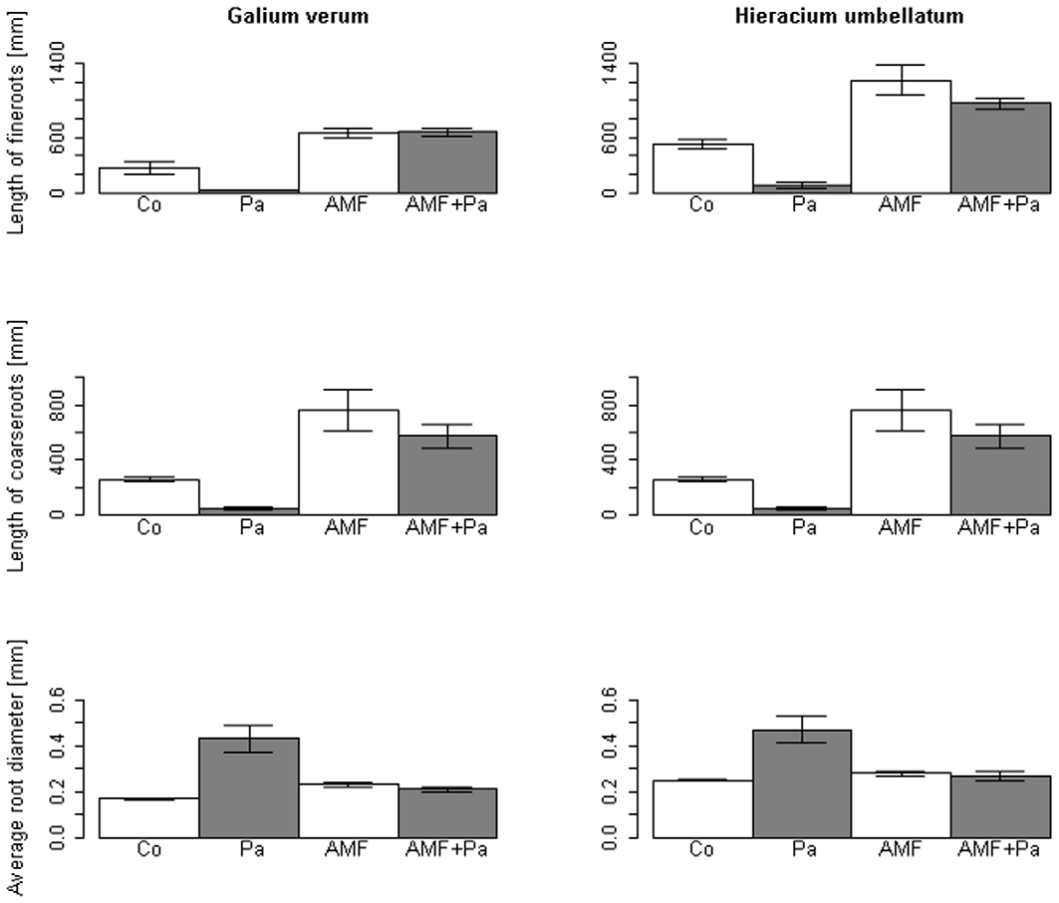
Effect of indigenous soil microbial treatment on the length of fine roots, length of coarse roots and average root diameter of *Galium verum* and *Hieracium umbellatum* in the four treatments (Co = Control, Pa = Pathogen community, AMF = AM fungal community, AMF + Pa). Error bars represent the standard error of the mean.

The root morphology followed similar patterns as total biomass. Fine root length and coarse root length had the highest values in the AM fungal treatments of both plant species (Fig. 2). We also found a significant decrease of these variables in the pathogen treatment compared to the controls for *H. umbellatum* (Table 4). For *G. verum* we only detected the pathogen effect for coarse root length, but a significant AM fungal × Pathogen interaction in all root length variables (Table 3). Furthermore, both plant species showed a significant increase in root diameter in the pathogen treatment by comparison with the AM fungal treatments and controls (Fig. 2).

### Fungal colonisation of roots

**Figure 3:**
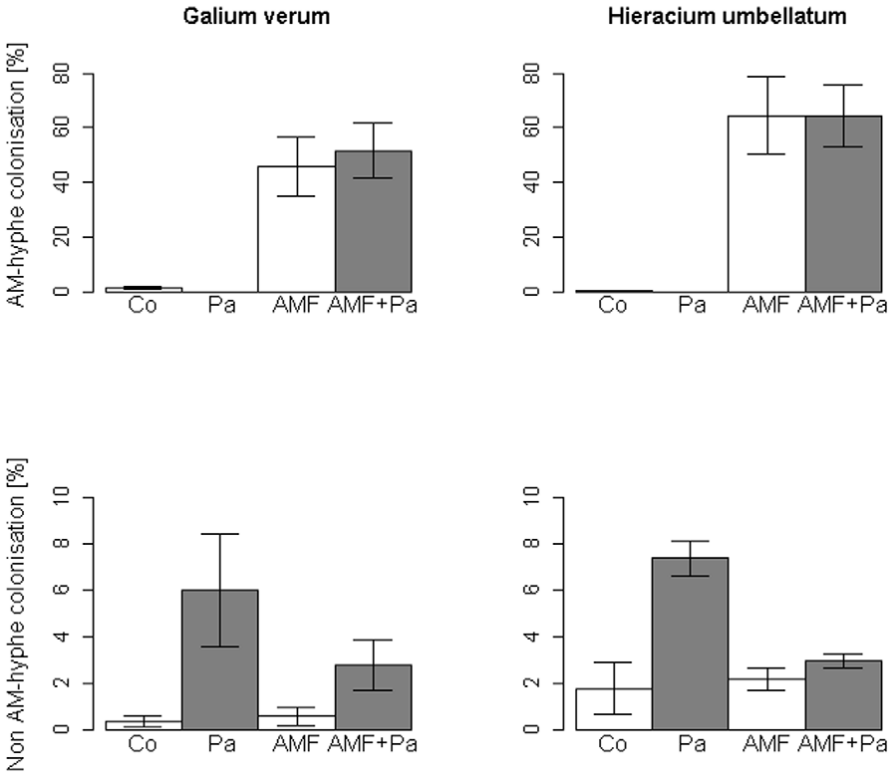
Percent root colonisation by AM fungal hyphae and non AM fungal hyphae in the roots of *Galium verum* and *Hieracium umbellatum* in the four treatments (Co = Control, Pa = Pathogen community, AMF = AM fungal community, AMF + Pa). Error bars represent the standard error of the mean.

We found AM fungal hyphae in AM fungal and AM fungi + Pathogen treatments in roots of both plants species, confirming that the AM fungal treatment was effective. AM fungal colonisation was similar in *G. verum* and *H. umbellatum* (Fig. 3). We found the highest colonisation by AM fungal hyphae in the two AM fungal treatments, which were significantly different from the non-AM fungal treatments (for *G. verum* df = 3, P = 0.0012 and *H. umbellatum* df = 3, P = 0.0012). Furthermore, we observed morphologically distinct colonization patterns within the roots, supporting the notion that plants were colonized by an assemblage of AM fungi. Only a few AM fungal-like hyphae were observed as background in the non-AM fungal treatments.

The extent of colonisation by non-AM fungal structures was low; these structures were found in all treatments (Fig. 3). Both plant species accumulated significantly more non-AM fungal structures in the pathogen treatment than in the non-mycorrhizal controls and AM fungal treatments (for *G. verum* df = 3, P = 0.014 and *H. umbellatum* df = 3, P = 0.007). We observed several hyphal types and other structures (spores, microsclerotia) that support the assessment that an assemblage of pathogens infected the plants. Furthermore, we detected a trend towards the reduction of non-AM fungal structures in the AM fungal treatment.

## Discussion

In this study we examined the interactions of indigenous AM fungal and pathogen/saprobe assemblages, both stemming from the same field site as the two plant species and the soil used. Overall, our results support the hypothesis that interactions between beneficial and pathogenic plant-soil microbial assemblages can influence the performance of certain plant species in a community.

AM fungi strongly increased the performance of both plant species compared to the non-AM fungal treatments. The effects of the pathogen/saprobe fraction were species dependent. While we found a significant decrease in the total biomass in presence of the pathogen/saprobe fraction compared to the controls and the sole AM fungal treatment in *H. umbellatum*, *G. verum* only negatively responded to the pathogen/saprobe fraction in absence of AM fungi. This indicates that the pathogen/saprobe fraction acted indeed as a pathogen treatment, as observed in previous experiments [20].

When AM fungi were present the negative effects of the pathogen/saprobe fraction were totally offset for *G. verum* whereas *H. umbellatum* also performed better than with the pathogen/saprobe fraction alone. Given that we found no significant interaction, this effect may not be caused by tolerance to the pathogen but rather be due to better nutrient supply in the presence of AM fungi. However, taken together, our results provide evidence for AM fungal mediated protection against the local pathogen/saprobe fraction.

Such pathogen protection is consistent with effects observed by Newsham et al. [12]; however, unlike these authors, we found an additional growth promoting effect caused by AM fungi, which was likely due to a better nutrient supply provided by different AM fungi in the assemblage, In our study we may have added different functional groups in the Glomeromycota, for which pathogen protection is suspected to be a phylogenetically-conserved trait [23], [24]. Some species within these AM fungal assemblages may have provided pathogen protection and some may have increased the nutrient status, which supports the hypothesis that functional complementarity could arise between different AM fungal species.

Belowground, the results obtained for root morphology were consistent with those observed aboveground. AM fungi increased the length of fine and coarse roots in both species. Although a highly branched root system should increase nutrient supply, it can also raise susceptibility against pathogens [25] and the plants benefit more from AM fungi via pathogen protection. However, we found a reduction in length of fine and coarse roots for both plant species in the pathogen treatment. This reduction might be due to direct damage to the root exodermis upon pathogen infection [26], thereby counteracting the AM fungal effect on root branching.

We consistently found an increase in root diameter in the pathogen treatment, which might be caused by the loss of fine roots either through pathogen attack or handling mistakes during harvest. Young and thinner roots are more susceptible against pathogens [26] and might have been destroyed.

We found pathogenic structures in both Pathogen and AMF + Pathogen treatments, but the presence of AM fungi reduced the pathogenic structures in the root tissue compared to the sole Pathogen treatment. However, we found pathogenic structures (i.e., conidia) in cells with AM fungi, which suggest that AM fungi might cause tolerance against pathogenic fungi in native plants. Due to the pathogen/saprobe fraction we used as pathogen treatment we cannot know if fungi alone caused the pathogenic effects or if other organisms played also an important role [27].

This is the first study to provide evidence for local AM fungal mediated plant pathogen protection by a resident AM fungal assemblage. We conclude that pathogen effects in natural ecosystems may be overestimated by studying single species and not considering diversity. In the field most plant species are associated with AM fungi, which can protect their host plants against pathogen attack under given conditions. Since such an important interaction between assemblages of beneficial and pathogenic organisms can take place, future research to understand factors controlling plant communities should focus on both organism groups and their interactions.

## Materials and Methods

### Ethics statement

All necessary permits were obtained for the described field studies at the nature protection area “Oderhänge Mallnow” from the Landesumweltamt Brandenburg, Referat Arten- und Biotopschutz, Potsdam, Germany.

### Field site

The study area from where seeds of both plant species and soil were collected is located in north-eastern Germany, approximately 120 km east of Berlin in the nature protection area “Oderhänge Mallnow” (52.4636°N, 14.4574°E, next to the small village of Mallnow), a Natura 2000 hotspot of biodiversity containing over 200 different plant species combining floral elements of both steppes and oceanic habitats. The site is a dry grassland habitat with *Adonis vernalis* and *Stipa capillata* as character species and part of the Adonido-Brachypodietum or rather Potentillo-Stipetum [28]. It is part of a large (60 km long and up to 20 km wide), formerly glacial, region with dry grassland habitats along the Oder river, called “Oderbruch”. The area is the most northerly-situated dry- and summer-warm region in Germany [28] and is characterized through its strongly continental climate with a mean annual precipitation around 500 mm and a mean annual temperature of 8.7°C [29].

### Soil and inoculum preparation

In June 2008 we collected soil from the grassland site. All the soil was sieved (2 mm), air dried and stored in boxes under cover until the experiment was set to start. A portion of approximately 100 kg was autoclaved (60 min at 121°C) for use as growth substrate for the plants. The soil contained 10.2 mg P kg^−1^, 30 mg K kg^−1^ and had a pH of 7.0.

For inoculum preparation soil cores (0–20 cm) were randomly collected at numerous locations (>20) spread across the entire grassland in January 2009. The AM fungal inoculum represented by a resident spore community was obtained from this field soil by wet sieving and sucrose gradient centrifugation using a method modified from Klironomos [20]: 1) soil cores were air dried and mixed to obtain a homogenous sample; 2) portions of 5 kg of soil (for a total of 40 kg) were suspended in 5 L water and passed through stacked 2 mm, 212 µm and 38 µm sieves; 3) AM fungal spores retained in the 38 µm sieve were surface sterilized for 1 min with 10% bleach, washed with tap water and collected in a beaker; 4) all spore collections were finally cleaned by suspending in 60% sucrose solution and centrifuging for 2 min at 960×g.

A pathogen/saprobe fraction (for simplicity we refer to this as ‘Pathogen’ treatment; but see discussion below) was obtained by passing a soil suspension, extracted from 2 kg of non-autoclaved soil, through a 20 µm sieve, which excludes AM fungal propagules. Although this fraction may contain non-pathogenic biota, comprised, for example, of saprobic or beneficial fungi or bacteria, this approach has been used as an effective method of isolating a community of pathogenic soil microorganisms able to negatively impact plant growth in an experimental system [20]. We spiked the pathogen fraction with a fungus isolated from plant roots randomly collected from our soil using selective media [30]. The isolated fungus was determined to be highly similar to *Ulocladium tuberculatum* by sequencing the ITS-region and doing a BLAST search in GenBank [National Center for Biotechnology Information (www.ncbi.nlm.nih.gov)]. Fungi in this genus are known as plant pathogens [31]. Controls were inoculated with an autoclaved AM spore suspension, pathogen community and cornmeal medium (20 min at 121°C).

### Seed collection and pre-germination

Seeds of *Galium verum* (Rubiaceae) and *Hieracium umbellatum* (Asteraceae), which are both common at the site, were collected at the end of the growing season in 2008. We chose these species because in addition to being abundant, they have similar root architecture despite being members of different plant families. The root systems of both species consist of a few coarse roots with branched fine roots. Root architecture is one of the most prominent traits in relation to pathogen susceptibility [25]. The seeds were surface sterilized with 70% ethanol, germinated in small plastic boxes with sterile soil from the field site and left to grow until the primary leaves had developed. Seedlings were transplanted to experimental units, which consisted of 4×20.5 cm ‘conetainers’ (Stuewe and Sons Inc., Corvallis, OR, USA).

### Experimental design

The experiment consisted of two crossed factors: presence/absence of AM fungi and pathogen, resulting in four treatment combinations per plant species. Containers were completely randomized on the greenhouse bench with 10 replicates per treatment for a total of 80 units. Plants were grown for three months with the AM fungal inoculum before adding the pathogen community. Since our goal was to test for AM fungal mediated pathogen protection, the gap between treatments was intended to let the AM fungal symbiosis establish first before challenging with the pathogen inoculum. We added 7 ml of a low P Hoagland's solution as fertilizer bi-weekly.

### Harvesting and post-harvest measurements

The experiment was harvested after six months of growth, which is equivalent to one growth season for these plant species in Central Europe. Due to poor survival rates in the treatments without AM fungi, treatment replication was reduced to five. The number of replicates in the AM fungal treatment was reduced by randomly choosing five individuals from all surviving replicates. Roots were separated from shoots, cleaned, and both parts were oven dried at 40°C for one week before being weighed for calculation of total biomass. Dried roots of all individuals of *G. verum* and *H. umbellatum* were re-hydrated, stained with ink-vinegar [32] and assessed for percentage of AM fungal colonisation [33]. We counted the number of AM and non-AM structures for 100 root intersections under the microscope using 200× magnification. For determining different colonization pattern of AM fungi we used the INVAM homepage (http://invam.caf.wvu.edu/). With this method we only captured fungal colonisation; we would have missed root colonisation by other microbiota like bacteria or viruses, which could be pathogenic as well. However, there is evidence that in grasslands, fungi appear to be often important root pathogens [20] and may contribute to plant species coexistence [34].

Furthermore we measured the length of fine (<2 mm) and coarse roots (>2 mm) and average root diameter with a flatbed scanner and the package WinRhizo (Régent Instruments Inc., 2007).

### Statistical analysis

All analyses were performed with the R software [35].

We started by calculating a MANOVA with the response variables total biomass, fine root length, coarse root length and root diameter. In fact, response variables showed different degrees of correlation, which requires a multivariate approach to protect posterior analysis on single variables. We then calculated single ANOVAs with two factors, “AM fungi” and “Pathogen” inoculation on total biomass, fine root length, coarse root length and root diameter for each species separately. Data were transformed as needed to meet ANOVA assumptions, however, untransformed values are reported in the figures. The Kruskal Wallis test was used to analyse percent root colonisation with AM and non-AM fungal structures within the treatments.
